# An overview of the design and methods for retrieving high-quality studies for clinical care

**DOI:** 10.1186/1472-6947-5-20

**Published:** 2005-06-21

**Authors:** Nancy L Wilczynski, Douglas Morgan, R Brian Haynes, the Hedges Team

**Affiliations:** 1 Health Information Research Unit, Department of Clinical Epidemiology and Biostatistics, McMaster University, Hamilton, Ontario, Canada; 2 Department of Clinical Epidemiology and Biostatistics, McMaster University, Hamilton, Ontario, Canada; 3 Michael G. DeGroote School of Medicine, McMaster University, Hamilton, Ontario, Canada

## Abstract

**Background:**

With the information explosion, the retrieval of the best clinical evidence from large, general purpose, bibliographic databases such as MEDLINE can be difficult. Both researchers conducting systematic reviews and clinicians faced with a patient care question are confronted with the daunting task of searching for the best medical literature in electronic databases. Many have advocated the use of search filters or "hedges" to assist with the searching process. The purpose of this report is to describe the design and methods of a study that set out to develop optimal search strategies for retrieving sound clinical studies of health disorders in large electronics databases.

**Objective:**

To describe the design and methods of a study that set out to develop optimal search strategies for retrieving sound clinical studies of health disorders in large electronic databases.

**Design:**

An analytic survey comparing hand searches of 170 journals in the year 2000 with retrievals from MEDLINE, EMBASE, CINAHL, and PsycINFO for candidate search terms and combinations. The sensitivity, specificity, precision, and accuracy of unique search terms and combinations of search terms were calculated.

**Conclusion:**

A study design modeled after a diagnostic testing procedure with a gold standard (the hand search of the literature) and a test (the search terms) is an effective way of developing, testing, and validating search strategies for use in large electronic databases.

## Background

The Clinical Hedges Study was designed with the objective of developing optimal search strategies to improve the retrieval of clinically relevant and scientifically sound study reports from large, general purpose, biomedical research bibliographic databases including MEDLINE, EMBASE, CINAHL, and PsycINFO. The search strategies were developed to 1) assist health care providers to do their own searches; 2) help reviewers of published evidence concerning health care interventions retrieve all relevant citations; 3) provide resources for librarians to help health care providers construct their own searches; and 4) provide input to the database producers about their indexing processes and the organization of their databases.

Data for the Clinical Hedges Study was collected throughout the year 2000 and continued into the year 2001. Database construction and analyses are ongoing with some of our pre-study results (calibration of the hand search) appearing in conference proceedings as early as 2001 [1]. Search strategies developed for use in MEDLINE have been published [2-9] but the study design and methods have not been fully detailed. We are in the process of publishing the search strategies developed for use in EMBASE [10], CINAHL, and PsycINFO. The current report provides full disclosure of the design and methods of the Clinical Hedges Study, including many details that could not be accommodated in other reports, to facilitate replication studies and comparisons with other approaches to bibliographic information retrieval.

## Methods/design

### Study design including data organization and programming

The methods and design of the Clinical Hedges Study are outlined in this paper and are illustrated in the Figure. The study design used to address the above mentioned objective was an analytic survey comparing hand searches of 170 journals in the year 2000 with retrievals from MEDLINE (161 of the 170 were indexed in MEDLINE in the year 2000), EMBASE (135 were indexed in EMBASE), CINAHL (75 were indexed in CINAHL), and PsycINFO (64 were indexed in PsycINFO) through the Ovid web gateway for candidate search terms and combinations. Candidate search terms and combinations were tested in the four electronic databases by treating the search terms as "diagnostic tests" for sound studies. Due to the size of these four electronic databases, it is not feasible to determine the total number of relevant citations in each database for a given search. The hand search of the literature for the 170 journals circumvents this problem as it provides a "gold standard" for a segment of the literature in the electronic database file, and an approximation for the operating characteristics of search strategies (i.e., sensitivity, specificity, precision, and accuracy).

There are two primary sources of data in the Clinical Hedges Study. The first source of data was generated from a hand search of the literature (the "gold standard"). The second source of data was generated from downloads of search terms and citation information from the four electronic databases (the "test"). After these two data sources were obtained, we created a database that contained the matched merged content from these sources. This merged database was used for the development and validation of search strategies. In the following sections we outline this process.

### Hand search data

To generate the hand search data six research assistants reviewed 170 journals titles for the year 2000. The 170 journal titles reviewed were chosen over several years in an iterative process based on hand search review of over 400 journals recommended by clinicians and librarians, Science Citation Index Impact Factors provided by the Institute for Scientific Information, recommendations by editors and publishers, and ongoing assessment of their yield of studies and reviews of scientific merit and clinical relevance. These journals include content for the disciplines of internal medicine (e.g., *Annals of Internal Medicine*), general medical practice (e.g., *BMJ*, *JAMA*, *Lancet*), mental health (e.g., *Archives of General Psychiatry*, *British Journal of Psychiatry*), and general nursing practice (e.g., *Nursing Research*) (list of journals provided by the authors upon request).

Each item (e.g., article, editorial, letter) in each issue of the 170 journals for the year 2000 was classified for article format (Table 1) and whether the content was of interest to human health care (Table 2). Original and review articles that were of interest to human health care were additionally classified for type of data presentation if a review article (Table 3), age of study participants (Table 4), purpose of the article (i.e., what question [s] is [are] the investigation addressing [Table 5]), and methodologic rigor for each of the purpose categories except for cost and qualitative studies and those articles classified as "something else" (Table 6). The methodologic criteria outlined in Table 6 are the same as those used for critically appraising articles for inclusion in 4 evidence-based medicine journals that our research group produced in 2000 (i.e., *ACP Journal Club, Evidence-Based Medicine, Evidence-Based Nursing*, and *Evidence-Based Mental Health*). Research staff were rigorously calibrated before the hand search of the 170 journal titles and inter-rater agreement for identifying the purpose of articles was 81% beyond chance (kappa statistic, 95% confidence interval [CI] 0.79–0.84). Inter-rater agreement for which articles met all scientific criteria was 89% (CI 0.78 to 0.99) beyond chance [1].

**Table 1:** Format categories

**Format type**	**Definition**
Original study	Any full text article in which the authors report first-hand observations.
Review article	Any full text article that was bannered 'review, overview, or meta-analysis' in the title or in a section heading, or it was indicated in the text of the article that the intention was to review, summarize, or highlight the literature on a particular topic.
General article	A general or philosophical discussion of a topic without original observation and without a statement that the purpose was to review a body of knowledge.
Case report	An original study or report that presented only individualized data.

**Table 2:** Interest to human health care

**Of interest**	**Definition**
Yes	Concerned with the understanding of health care in humans; anything that will have an effect on the patient/subject.
No	Not concerned with the understanding of health care in humans; anything that will not have an effect on the patient/subject (e.g., studies that describe the normal development of people; basic science; studies involving animals; gender and equality studies in the health profession; or studies looking at research methodology issues).

**Table 3:** Categories of data presentation in review articles

**Type of data presentation**	**Definition**
Individual patient data	Individual patient data was used in a meta-analysis.
Meta-analysis	The reported summary data were pooled from relevant studies.
Overview	A general discussion of the reviewed studies with no attempt to quantitatively combine the results.

**Table 4:** Age categories of ≥ 50% of study participants

**Category**	**Definition**
Fetus	Fetus
Newborn	Birth to 1 month
Infant	> 1 month to < 24 months
Preschool	2 years to < 6 years
Child	6 years to < 13 years
Adolescent	13 years to < 19 years
Adult	19 years to < 45 years
Middle age	45 years to < 65 years
Aged	65 years to < 80 years
Aged 80	≥ 80 years
ND	Age of study participants was non-discernible

**Table 5:** Purpose categories

**Purpose type**	**Definition**
Etiology	Content pertained directly to determining if there was an association between an exposure and a disease or condition. The question is "What causes people to get a disease or condition?"
Prognosis	Content pertains directly to the prediction of the clinical course or the natural history of a disease or condition with the disease or condition existing at the beginning of the study.
Diagnosis	Content pertains directly to using a tool to arrive at a diagnosis of a disease or condition.
Treatment	Content pertains directly to an intervention for therapy (including adverse effects studies), prevention, rehabilitation, quality improvement, or continuing medical education.
Cost	Content pertains directly to the costs or financing or economics of a health care issue.
Economics	Content pertains directly to the economics of a health care issue.
Clinical Prediction Guide	Content pertains directly to the prediction of some aspect of a disease or condition.
Qualitative	Content relates to how people feel or experience certain situations, and data collection methods and analyses are appropriate for qualitative data.
Something Else	Content of the study does not fit any of the above definitions.

**Table 6:** Methodologic rigor

**Purpose category**	**Methodologic rigor**
Etiology	Observations concerned with the relationship between exposures and putative clinical outcomes; Data collection is prospective; Clearly identified comparison group(s); Blinding of observers of outcome to exposure.
Prognosis	Inception cohort of individuals all initially free of the outcome of interest; Follow-up of ≥ 80% of patients until the occurrence of a major study end point or to the end of the study; Analysis consistent with study design.
Diagnosis	Inclusion of a spectrum of participants; Objective diagnostic ("gold") standard OR current clinical standard for diagnosis; Participants received both the new test and some form of the diagnostic standard; Interpretation of diagnostic standard without knowledge of test result and vice versa; Analysis consistent with study design.
Treatment	Random allocation of participants to comparison groups; Outcome assessment of at least 80% of those entering the investigation accounted for in 1 major analysis at any given follow up assessment; Analysis consistent with study design.
Economics	Question is a comparison of alternatives; Alternative services or activities compared on outcomes produced (effectiveness) and resources consumed (costs); Evidence of effectiveness must be from a study of real patients that meets the above-noted criteria for diagnosis, treatment, quality improvement, or a systematic review article; Effectiveness and cost estimates based on individual patient data (micro-economics); Results presented in terms of the incremental or additional costs and outcomes of one intervention over another; Sensitivity analysis if there is uncertainty.
Clinical Prediction Guide	Guide is generated in one or more sets of real patients (training set); Guide is validated in another set of real patients (test set).
Review articles	Statement of the clinical topic; Explicit statement of the inclusion and exclusion criteria; Description of the methods; ≥ 1 article must meet the above noted criteria.

Hand search data were recorded on a paper based data collection form that was compatible with an optical mark and character recognition system called Teleform (Cardiff Software Publishing, Bozeman MT). The data collection form was designed using Teleform Designer. On each form data entry fields were available for the journal name, volume, issue and publication date, as well as a tabular data collection area to record the classification of individual items in the journal (e.g., article, letter, news item – 16 records per sheet). Multiple pages of the form could apply to one issue of a journal. Alphanumeric data fields were encoded in Teleform "comb" fields, where each hand printed block character had to be drawn within a fixed assigned space. A variety of strategies were used to maximize the reliability of the optical character/mark recognition abilities of Teleform. As many of the data fields as possible were either multiple choice or numeric. For fields containing text, research staff were trained in optimal letter formation and were required to use designated pre-tested pens. A mechanism was also established for correcting data entry errors.

All data collection forms were scanned with a Hewlett Packard 610cxi Scanjet scanner using Teleform Reader and the interpretation of the form was verified using Teleform Verifier. Scripts were written in a limited form of Visual Basic for Applications to perform basic validation on the incoming data while being interpreted by Teleform Reader. Data entry staff performed a data verification step by comparing the scanned image of the form alongside the Teleform interpretation. The staff were given the opportunity to accept the interpretation or to correct it. The original data collection sheets and/or the researcher who classified the material may have been consulted to make corrections. Tests to determine data entry error rates were conducted and an overall error rate of 0.01% was found.

After data entry and data verification using Teleform, hand search data were exported to a Microsoft (MS) Access database. The hand search data was split into two MS Access tables, one containing journal information and the other containing article information. The two tables were linked on a key field.

The hand search database volume was extensive with 11 data fields recorded for a collection of 60,330 articles in 170 publications.

### On-line data

The data acquired from the on-line database were matching information (i.e., journal name, issue, and volume; first author's last name; title of the article; and first page number of the article), and the results of executing search terms in the on-line database. To generate the second source of data, the on-line data, it was necessary to construct a comprehensive set of search terms. We began a list of index terms and textwords for each of the four electronic databases, MEDLINE, EMBASE, CINAHL, and PsycINFO, and then sought input from clinicians and librarians in the United States and Canada through interviews of known searchers, requests at meetings and conferences, and requests to the National Library of Medicine. Individuals were asked what terms or phrases they used when searching for studies of causation, prognosis, diagnosis, treatment, economics, clinical prediction guides, reviews, costs, and of a qualitative nature when using these databases. For instance, for MEDLINE, terms could be from Medical Subject Headings (MeSH), including publication types (pt), and subheadings (sh), or could be textwords (tw) denoting methodology in titles and abstracts of articles. We compiled a list of 5,345 terms for MEDLINE of which 4,862 were unique and 3,870 returned results (list of terms tested provided by the authors upon request). For EMBASE we compiled a list of 5,385 terms of which 4,843 were unique and 3,524 returned results (list of terms tested provided by the authors upon request). For CINAHL we compiled a list of 5,020 unique search terms of which 3,110 returned results (list of terms tested provided by the authors upon request). For PsycINFO we compiled a list of 4,985 unique search terms of which 2,583 returned results (list of terms tested provided by the authors upon request). Index terms varied by electronic database whereas the same list of textwords were tested in each of the electronic databases.

Since the primary goal of the Clinical Hedges Study was to deliver search strategies which could be used by clinicians and researchers to locate the best quality published research specific to their interests it was required that the data be gathered via the same user interface that end users would use. Thus, raw performance data for individual search terms were downloaded via Ovid. Because of the volume of terms and their combinations, we automated the submission of terms using a telnet connection.

Ovid provides a simulated Graphical User Interface (GUI) through telnet using a simulated Dec VT-100 terminal interface. To handle a series of exchanges an open loop automation scheme was used. This consisted of a script-reading program written in Visual Basic for Applications, which passed keystroke data that mimicked what a user would enter into a commercially available telnet program. The script reader retrieved specific details from internally derived reference tables such as journal names (the 170 journals that were hand searched) and search terms (text of all search terms compiled for each of the four electronic databases) and inserted this information into the script through a parameter substitution scheme. To retrieve search term data for the Clinical Hedges Study, we "ANDed" each search term with a strategy saved on Ovid, which comprised our reading list of 170 journals published in the year 2000.

We used MS Outlook to recover the requested data via e-mail. Filters and dedicated "pst" files were set up to handle e-mail retrieval from a research project e-mail account. An "autosave" program saved the e-mail messages in individual text files for automated processing. A "downloads" program read the saved text files and entered the data into the appropriate MS Access tables. These scripts were stored in an MS Access database along with the program, and were organized in tables where keystroke sequences formed individual commands, which could be timed. The sequences of commands were grouped according to their function in the process of connecting to the on-line service (Ovid) or gathering data from it.

### Matching hand search and on-line data

The approach to matching the hand search data records to the on-line data was undertaken in a two-stage process. First, a minimal set of information was retrieved from the on-line source, organized by journal title, which was used to link hand search data. At this stage, the linking software only attempted to match the hand-coded journal, volume, and issue information from the hand search data to a similarly hand-coded field retrieved from the on-line source. Later, a more complete set of matching information was retrieved from the on-line source, including a unique identifier for the index journals, article titles, authors, abstracts, indexing terms, and publication types. These data were organized by journal and index.

The matching algorithm was initially conservative requiring 100% certainty to establish a match. An "unmatched" report was subsequently generated which was used to refine the algorithm. For each of the four electronic databases approximately 95% of the records could be matched through an automated process. The remaining unmatched records were processed manually.

It should be noted here that there was not a one-to-one relationship between individual items entered in the hand search database and items recovered from the on-line database because an individual article could be determined to serve more than one purpose in the context of the clinical HEDGES study. Thus, an article could be a "review" (one format) about "diagnosis" and "therapy", which were additional purposes.

After extensive attempts, a small fraction of the hand-search items failed to be matched to citations in each of the four electronic databases and a small number of citations downloaded from each of the four electronic databases failed to be match to the hand-search data. As a conservative approach, unmatched citations that were detected by a given search strategy were included in cell 'b' of the analysis table (Table 7) leading to slight underestimates of the precision, specificity, and accuracy of the search strategy. Similarly, unmatched citations that were not detected by a search strategy were included in cell 'd' of the table (Table 7), leading to slight overestimates of the specificity and accuracy of the strategy.

**Table 7:** Formulae for calculating the sensitivity, specificity, precision, and accuracy of searches for detecting sound clinical studies

		Manual Review (Hand search)	
		
		Meets Criteria	Does Not Meet Criteria
Search Terms	Detected	a	b
	Not detected	c	d
		a + c	b + d

a = true positives, articles found by the search term meet the criteria for purpose category (e.g., treatment) and methodologic rigor (i.e., "pass")

b = false positives, articles found by the search term do not meet the criteria for purpose category and methodologic rigor

c = false negatives, articles not found by the search term but did meet the criteria for purpose category and methodologic rigor

d = true negatives, articles not found by the search term and did not meet the criteria for purpose category and methodologic rigor

Sensitivity = a/(a+c); Precision = a/(a+b); Specificity = d/(b+d);

Accuracy = (a+d)/(a+b+c+d).

All articles classified during the manual review of the literature, n = (a+b+c+d).

### Computations

After the hand search and on-line data were matched the merged data file was prepared for deriving computations. At this point, other than the unique identifiers from both data sources (hand search and on-line data), none of the other matching information (journal name, journal issue, etc) was relevant to the computations. This extraneous data was, therefore, removed from the tables that were used to compute the performance of the search terms, as this information was already stored in a journal table within MS Access.

We determined the sensitivity, specificity, precision, and accuracy of each single term and combinations of terms using an automated process. The formulae for calculating these statistics are shown in Table 7. Sensitivity for a given topic (e.g., articles that are classified as original treatment studies that "pass" methodologic rigor) is defined as the proportion of high quality articles for that topic that are retrieved; specificity is the proportion of low quality articles not retrieved; precision is the proportion of retrieved articles that are of high quality; and accuracy is the proportion of all articles that are correctly classified.

Once the performance parameters of individual search terms were computed, it was possible to select individual terms for the construction of search strategies. In the Clinical Hedges Study, we had a collection of over 4,800 unique search terms for MEDLINE and EMBASE, with up to 1,200 of them returning results for a particular purpose category (e.g., treatment, prognosis). A rather simple estimation of computation time required, based on single term calculations and some preliminary two-term calculation runs, indicated that the time to compute search strategies using all of the available terms and an arbitrary limit on the number of terms in combinations, could amount to literally hundreds of years of computing time with equipment available at the time. Many of the combinations of search terms could be predicted to perform poorly as they contained individual terms that had poor performance in terms of returning very few true positives, or returning far too many false positives (for definition of terms see Table 7). Thus, for MEDLINE and EMBASE, only individual search terms with sensitivity > 25% and specificity > 75% for a given purpose category were incorporated into the development of search strategies that included 2 or more terms. All combinations of terms used the Boolean OR, for example, "predict.tw. OR survival.sh.". For the development of multiple-term search strategies to either optimize sensitivity or specificity, we tested all 2-term search strategies with sensitivity at least 75% and specificity at least 50%. For optimizing accuracy, 2-term search strategies with accuracy > 75% were considered for multiple-term development. These criteria were relaxed somewhat for CINAHL and PsycINFO since the number of terms returning results were fewer. Individual search terms with sensitivity ≥ 10% and specificity ≥ 10% for a given purpose category were incorporated into the development of search strategies that included 2 or more terms. All possible "ORed" two-and three-term combination of terms for each of the purpose categories were derived and tested through an automated iterative process. Our target through combining search terms was to optimize each of sensitivity, specificity, and accuracy.

In addition to developing search strategies using the Boolean approach described above, we also evaluated the potential for improving performance using logistic regression. Two approaches were taken. First, we took the top performing Boolean search strategies and ORed additional terms to these base strategies using stepwise logistic regression. The level of significance for entering and removing search terms from the model was 0.05. Adding terms to the model stopped when the increase in the area under the ROC curve was < 1%. Second, we developed search strategies from scratch with stepwise logistic regression using these same cut off values. Both logistic regression approaches were compared with the Boolean approach to search strategy development when developing strategies for treatment articles and prognostic articles for MEDLINE. Treatment and prognosis were chosen because they represented the best and the worst cases for MEDLINE search strategy performance. For both purpose categories the logistic regression approaches to developing search strategies did not improve performance compared with search strategies developed using the Boolean approach described above. We also found that when strategies were developed in 60% of the database and validated in the remaining 40% (a random allocation method was used to assign individual items to the development or validation datasets) there were no statistical differences in performance. Thus, for subsequent purpose categories and databases, the Boolean approach was used for search strategy development and search strategies were developed using all records in the database.

Search strategies developed for use in MEDLINE have been translated for use in PubMed by staff of the National Library of Medicine, and compared for performance by the senior author (RBH).

## Discussion

A study design modeled after a diagnostic testing procedure with a gold standard (the hand search of the literature) and a test (the search terms) is an effective way of developing, testing, and validating search strategies for use in large electronic databases.

Additional research is underway in search strategy development including testing the strategies developed through this research, when combined with disease content terms, and when combined with terms using the Boolean "AND" and/or "NOT".

## Competing interests

The author(s) declare that they have no competing interests.

## Authors' contributions

NLW and RBH prepared grant submissions in relation to this project and supplied intellectual content to the collection and analysis of the data. NLW participated in the data collection and all authors were involved in data analysis. All authors drafted, commented on and approved the final manuscript.

## The Hedges Team

Includes Angela Eady, Brian Haynes, Chris Cotoi, Susan Marks, Ann McKibbon, Doug Morgan, Cindy Walker-Dilks, Stephen Walter, Stephen Werre, Nancy Wilczynski, and Sharon Wong.

**Figure 1:**
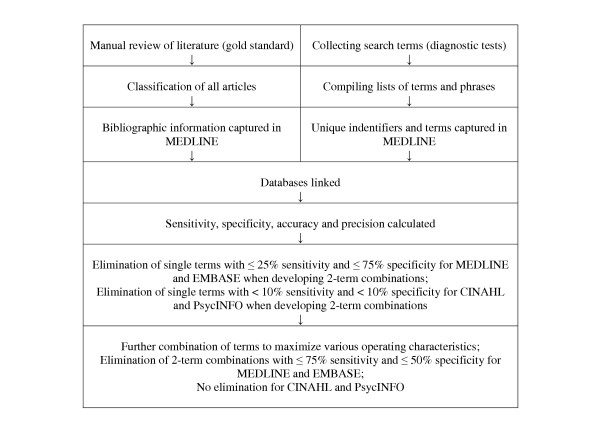
Steps in data collection.

## Pre-publication history

The pre-publication history for this paper can be accessed here:



## References

[B1] Wilczynski NL, McKibbon KA, Haynes RB (2001). Enhancing retrieval of best evidence for health care from bibliographic databases: calibration of the hand search of the literature. Medinfo.

[B2] Wilczynski NL, Haynes RB (2003). Developing optimal search strategies for detecting clinically sound causation studies in MEDLINE. Proc AMIA Symp.

[B3] Wong SS, Wilczynski NL, Haynes RB, Ramkissoonsingh R (2003). Developing optimal search strategies for detecting sound clinical prediction studies in MEDLINE. Proc AMIA Symp.

[B4] Haynes RB, Wilczynski NL (2004). Optimal search strategies for retrieving scientifically strong studies of diagnosis from MEDLINE: analytical survey. BMJ.

[B5] Wilczynski NL, Haynes RB (2004). The Hedges Team. Developing optimal search strategies for detecting clinically sound prognostic studies in MEDLINE. BMC Medicine.

[B6] Wong SS, Wilczynski NL, Haynes RB (2004). Developing Optimal Search Strategies for Detecting Clinically Relevant Qualitative Studies in MEDLINE. Medinfo.

[B7] Montori VM, Wilczynski NL, Morgan D, Haynes RB, Hedges Team (2005). Optimal search strategies for retrieving systematic reviews from MEDLINE: analytical survey. BMJ.

[B8] Haynes RB, McKibbon KA, Wilczynski NL, Walters SD, Werre SR, Hedges Team (2005). Optimal search strategies for retrieving scientifically strong studies of treatment from MEDLINE: an analytical survey. BMJ.

[B9] Wilczynski NL, Haynes RB, Lavis JN, Ramkissoonsingh R, Arnold-Oatley AE, HSR Hedges team (2004). Optimal search strategies for detecting health services research studies in MEDLINE. CMAJ.

[B10] Haynes RB, Kastner M, Wilczynski NL, Hedges Team (2005). Developing optimal search strategies for detecting clinically sound and relevant causation studies in EMBASE. BMC Med Inform Decis Mak.

